# FAST breast magnetic resonance imaging: a new approach for breast cancer screening?

**DOI:** 10.31744/einstein_journal/2022AO0073

**Published:** 2022-07-08

**Authors:** Vanessa Sales Vilar, Andressa Inácio Gomes, Érica Elisângela Françolin Federicci, Renato Leme de Moura Ribeiro, Mônica Akahoshi Rudner, Ana Cláudia Silveira Racy

**Affiliations:** 1 Hospital Israelita Albert Einstein São Paulo SP Brazil Hospital Israelita Albert Einstein, São Paulo, SP, Brazil.

**Keywords:** FAST, Breast, Magnetic resonance imaging, Mass screening

## Abstract

**Objective:**

To develop an abbreviated breast magnetic resonance imaging protocol (FAST) and to compare it with the complete protocol (FULL) to determine its diagnostic accuracy for detecting malignant or suspicious lesions (BI-RADS 4, 5 and 6) and the time required for image interpretation using BI-RADS categorization.

**Methods:**

Retrospective study with 100 consecutive women who underwent breast magnetic resonance imaging between January and February 2014. All patients were submitted to a complete breast magnetic resonance imaging protocol, which was then compared with an abbreviated protocol (pre-contrast sequence, second post-contrast sequence and subtraction of pre- from post-contrast images).

**Results:**

Of 100 patients, 4 were classified as BI-RADS 5 or 6 and 16 as BI-RADS 4. In these 20 patients, there was full agreement among readers regarding the final BI-RADS categorization in both (FAST and FULL) protocols.

**Conclusion:**

The FAST protocol reduces interpretation time without compromising the accuracy of the method for detection of malignant or suspicious lesions.

## INTRODUCTION

Mammography is the gold standard method for breast cancer screening, with randomized studies showing a reduction in mortality in screened patients. However, this imaging modality has some limitations, especially in women with high-density breast tissue, and may lead to overdiagnosis of clinically non-relevant lesions and underdiagnosis of clinically relevant lesions. Breast magnetic resonance imaging (MRI) has become an essential tool for breast cancer diagnosis and staging and is a highly sensitive method. However, MRI requires the use of contrast agents and is time consuming and often uncomfortable for the patient, not to mention the long reading time for the radiologist. With the high sensitivity for detection of high-grade invasive and *in situ* lesions in mind, an abbreviated breast MRI (FAST) protocol, based primarily on the contrast phase, has been proposed as a screening method with the purpose of reducing scan cost and time (up to 3 minutes) as well as the time required by the radiologist to read and interpret images, with good results according to some studies.^(1,2)^

This study was designed to investigate whether the FAST MRI protocol is an effective method for breast cancer screening. The aims were to compare the FAST and FULL protocols in women undergoing breast MRI for different reasons and to determine whether the FAST protocol is as sensitive and specific as the FULL protocol.

A few studies, most of them retrospective, have compared FAST and FULL MRI protocols in specific populations, such as women with dense breasts or at high risk of developing breast cancer, to test sensitivity and specificity of each method.^(3)^

This study aims to test both protocols in the general female population, a critical step for assessment and validation of this new protocol in our population.

## OBJECTIVE

To develop an abbreviated breast magnetic resonance imaging protocol (FAST) and to compare this protocol with the complete protocol (FULL) to determine its diagnostic accuracy for detecting malignant or suspicious lesions (BI-RADS 4, 5 and 6) and the time required for image interpretation using BI-RADS categorization.

## METHODS

**Figure 1:**
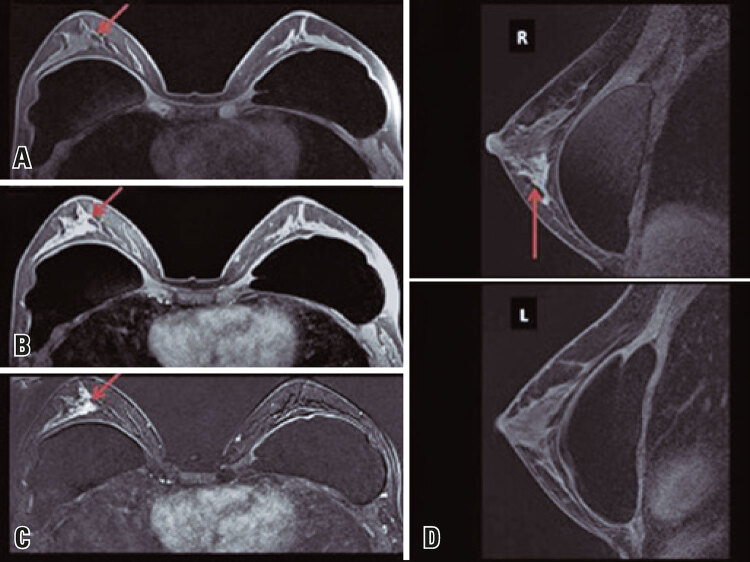
Female, 41 years old, family history of breast cancer. Breast magnetic resonance imaging revealed a focal area of heterogeneous and asymmetric enhancement measuring 3.3 x 4.0 x 2.3cm at the union of the lower quadrants of the right breast (red arrows), classified as BI-RADS 4. (A) Fat-suppressed pre-contrast T1 image; (B) Second post-contrast T1 sequence; (C) Subtraction image (post-contrast - pre-contrast); (D) Sagittal reconstruction of post-contrast image

Source: Vilar VS, Gomes AI, Cubo JV, Racy AC, Ribeiro RL, Francolin E. Fast breast MRI: a new approach for breast cancer screening? In: 25^th^ European Congress of Radiology; 2019 Feb 27-Mar 3; Vienna. Annals. Vienna: European Society of Radiology; 2019 [cited 2021 Sep 22]. Available from: https://epos.myesr.org/poster/esr/ecr2019/C-1490^(4)^

**Figure 2:**
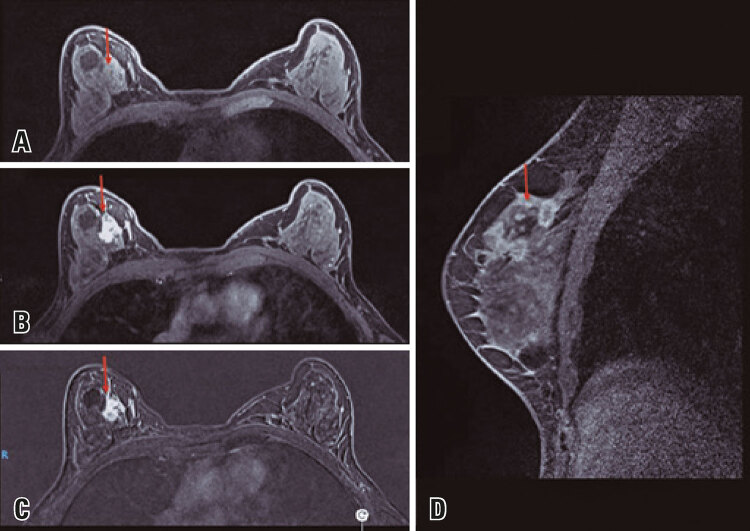
Female, 48 years old, family history of breast cancer. Magnetic resonance imaging revealed irregular and spiculated masses in the right breast, with heterogeneous enhancement and type III dynamic curve (red arrows), consistent with ultrasonographic and mammographic findings described on the same date and classified as BI-RADS 5. (A) Fat-suppressed pre-contrast T1 image; (B) Second post-contrast T1 sequence; (C) Subtraction image (post-contrast - pre-contrast); (D) Sagittal reconstruction of post-contrast image

Source: Vilar VS, Gomes AI, Cubo JV, Racy AC, Ribeiro RL, Francolin E. Fast breast MRI: a new approach for breast cancer screening? In: 25^th^ European Congress of Radiology; 2019 Feb 27-Mar 3; Vienna. Annals. Vienna: European Society of Radiology; 2019 [cited 2021 Sep 22]. Available from: https://epos.myesr.org/poster/esr/ecr2019/C-1490^(4)^

**Figure 3:**
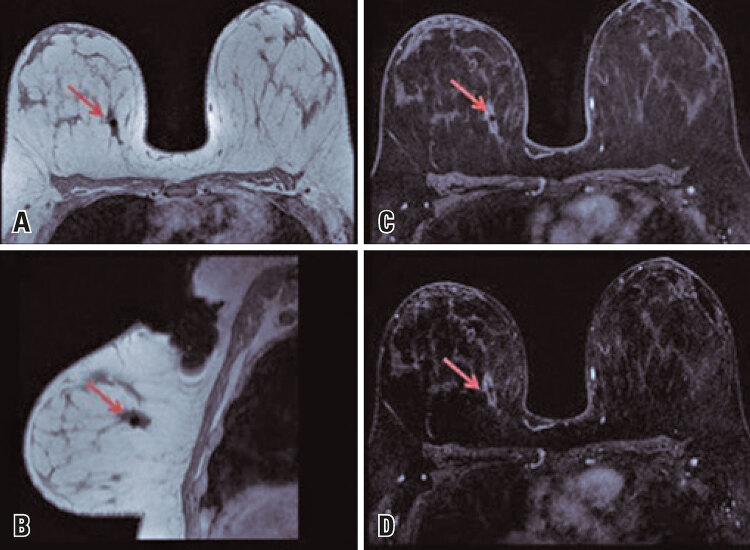
Female, 34 years old, with a palpable mass in the right breast. Biopsy was positive for malignant lesion. Follow-up magnetic resonance imaging after neoadjuvant chemotherapy revealed a post vacuum-assisted biopsy clip contained within an irregular and spiculated mass with peripheral heterogeneous enhancement and measuring 2.5 x 1.2 x 1.1cm. This mass was located in the posterior third of the upper inner quadrant of the right breast (red arrows) and suggested partial response to chemotherapy (classified as BI-RADS 6). (A) Pre-contrast T1 image without fat saturation; (B) Pre-contrast T1 sagittal reconstruction; (C) Second post-contrast T1 sequence; (D) Subtraction image (post-contrast - pre-contrast)

Source: Vilar VS, Gomes AI, Cubo JV, Racy AC, Ribeiro RL, Francolin E. Fast breast MRI: a new approach for breast cancer screening? In: 25^th^ European Congress of Radiology; 2019 Feb 27-Mar 3; Vienna. Annals. Vienna: European Society of Radiology; 2019 [cited 2021 Sep 22]. Available from: https://epos.myesr.org/poster/esr/ecr2019/C-1490^(4)^

This is a retrospective study based on the analysis of 100 breast MRI scans performed at our institution between January and February 2014. All patients were submitted to the service’s standard protocol (FULL) consisting of T1-weighted sequences, a dynamic pre-contrast T2 sequence with 4 post-contrast phases plus 1 late phase and lasting 20 minutes on average. This protocol was compared with FAST protocol, which includes only the pre-contrast sequence, the second post-contrast sequence (Figures 1, 2 and 3)^(4)^ and the analysis of image subtraction, with an average examination time of 3 minutes.^(5)^ Both (FAST and FULL) protocols were analyzed by at least two radiologists with more than 10 years of experience in breast imaging, who classified the exams as positive (with relevant imaging findings) or negative (no relevant imaging findings) and described positive findings using the BI-RADS lexicon.^(6)^ Inter-reader and inter-protocol variability regarding BI-RADS classification and diagnostic accuracy were examined.

This study was approved by the Research Ethics Committee of *Hospital Israelita Albert Einstein* (HIAE), (# 4.770.355, CAAE: 39120920.7.0000.0071).

## RESULTS

**Figure 4:**
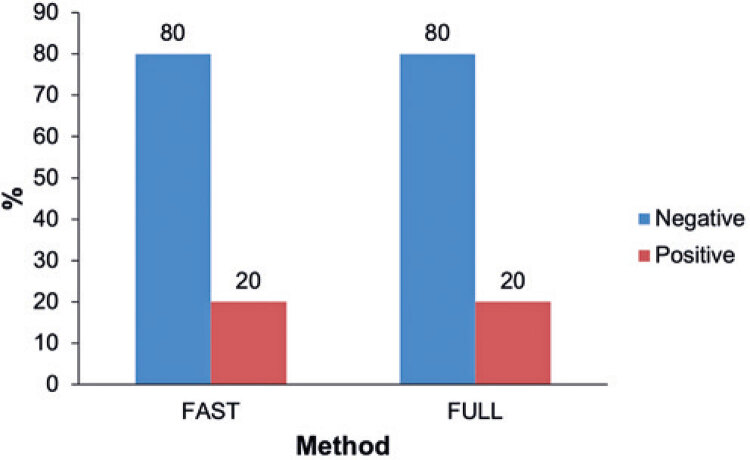
Representative graph of the agreement rate between both methods (FAST and FULL MRI protocols) with regard to detection of malignant or suspicious lesions (classified as positive - red columns)

Source: Vilar VS, Gomes AI, Cubo JV, Racy AC, Ribeiro RL, Francolin E. Fast breast MRI: a new approach for breast cancer screening? In: 25^th^ European Congress of Radiology; 2019 Feb 27-Mar 3; Vienna. Annals. Vienna: European Society of Radiology; 2019 [cited 2021 Sep 22]. Available from: https://epos.myesr.org/poster/esr/ecr2019/C-1490^(4)^

Of 100 patients, 4 were classified as BI-RADS 5 or 6 and 16 as BI-RADS 4. In 20 patients there was full agreement (100%) among readers concerning the final BI-RADS categorization in the FAST and FULL protocols. In spite of BI-RADS inter-reader agreement, in 4 out of 20 patients with final BI-RADS 4, 5 or 6 categorization the FAST protocol failed to detect one or more lesions identified in the FULL protocol. However, since these lesions were not suspected of malignancy, no significant lesions were missed when the FAST protocol was used, with an agreement rate of 100% between methods (*i.e.,* FULL and FAST protocols) (Figure 4).^(4)^

**Figure 5:**
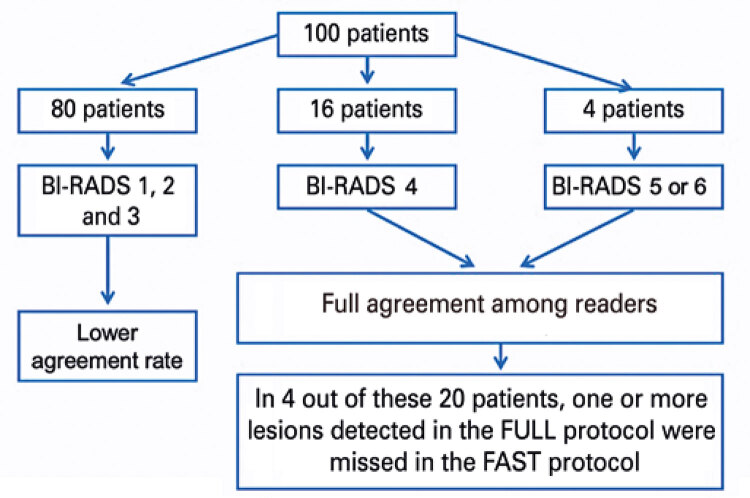
Representative scheme of the agreement rate among readers with regard to final BI-RADS categorization in the FULL and FAST MRI protocols

Source: Vilar VS, Gomes AI, Cubo JV, Racy AC, Ribeiro RL, Francolin E. Fast breast MRI: a new approach for breast cancer screening? In: 25^th^ European Congress of Radiology; 2019 Feb 27-Mar 3; Vienna. Annals. Vienna: European Society of Radiology; 2019 [cited 2021 Sep 22]. Available from: https://epos.myesr.org/poster/esr/ecr2019/C-1490^(4)^

As to BI-RADS 1, 2 and 3, agreement rates were lower mainly due to the presence or absence of small, purely cystic lesions (Figure 5).^(4)^ Use of the abbreviated protocol significantly reduced interpretation time: the average interpretation time of the abbreviated protocol (FAST) was 21 minutes, whereas the average interpretation time of the complete protocol (FULL) was 59 minutes.

## DISCUSSION

Mammography is the gold standard method for breast cancer screening. However, this imaging modality has some limitations and may lead to overdiagnosis of prognostically unimportant lesions and underdiagnosis of relevant cancers.^(5)^

Magnetic resonance imaging has become an essential tool in breast cancer diagnosis and staging and is a highly accurate, effective and widely accepted method for screening women at high risk. The formal indications for breast MRI based screening are women with calculated lifetime risk of breast cancer higher than 20%, women with genetic predisposition to breast cancer (BRCA mutations) and women with a history of chest irradiation at a young age. Magnetic resonance imaging is also recommended for women with a personal history of breast cancer, dense breast tissue or breast cancer diagnosis before the age of 50 years.^(6,7)^

Breast MRI has the highest sensitivity in cancer detection compared to other supplemental screening modalities such as ultrasonography, mammography, or even digital breast tomosynthesis. However, it has low specificity, high costs and long image acquisition time and there is a paucity of data in women with average to intermediate risk.^(8)^

Abbreviated breast MRI was proposed in an effort to reduce scan costs and time (up to 3 minutes), but especially due to the reduction of the time required for image reading and interpretation by the radiologist.^(9)^

With this scenario in mind, this study set out to investigate whether an abbreviated breast MRI protocol would be an effective tool for breast cancer screening in women other than those at high risk of developing breast cancer (*i.e*., general female population).

Although our findings were encouraging, this study has some limitations: the sample was small, there was no disagreement among methods or readers and the study was retrospective. A larger sample of patients is needed to confirm whether the FAST protocol is effective for breast cancer screening. It would be also interesting to include T2-weighted sequences to avoid false positives in cases with cystic lesions.

A few (mostly retrospective) studies have compared FAST and FULL MRI protocols for screening of specific populations, such as women with dense breasts or at high risk of developing breast cancer, to test the sensitivity and specificity of each method.^(10,11)^ Such studies are crucial for evaluation and validation of this new (FAST MRI) protocol.

## CONCLUSION

The abbreviated breast magnetic resonance imaging protocol (FAST) reduces image interpretation time without compromising the accuracy for detecting malignant or suspicious lesions and may be an effective tool for breast cancer screening in specific populations.
